# Use of a Th1 Stimulator Adjuvant for Vaccination against *Neospora caninum* Infection in the Pregnant Mouse Model

**DOI:** 10.3390/pathogens2020193

**Published:** 2013-03-27

**Authors:** Thierry Monney, Denis Grandgirard, Stephen L. Leib, Andrew Hemphill

**Affiliations:** 1 Institute of Parasitology, Vetsuisse Faculty, University of Berne, Länggass-Strasse 122, CH-3012 Berne, Switzerland; 2 Institute of Infectious Diseases, University of Berne, Friedbühlstrasse 51, CH-3010 Berne, Switzerland; E-Mails: denis.grandgirard@ifik.unibe.ch (D.G.); stephen.leib@ifik.unibe.ch (S.L.L.); 3 Biology Division, Spiez Laboratory, Swiss Federal Office for Civil Protection, CH-3700 Spiez, Switzerland

**Keywords:** *Neospora caninum*, vaccination, recombinant antigen, pregnancy, vertical transmission, cytokines, mouse model, abortion

## Abstract

Vertical transmission from an infected cow to its fetus accounts for the vast majority of new *Neospora caninum* infections in cattle. A vaccine composed of a chimeric antigen named recNcMIC3-1-R, based on predicted immunogenic domains of the two microneme proteins NcMIC1 and NcMIC3, the rhoptry protein NcROP2, and emulsified in saponin adjuvants, significantly reduced the cerebral infection in non-pregnant BALB/c mice. Protection was associated with a mixed Th1/Th2-type cytokine response. However, the same vaccine formulation elicited a Th2-type immune response in pregnant mice and did not prevent vertical transmission or disease, neither in dams nor in offspring mice. In this study, an alternative vaccine formulation containing recNcMIC3-1-R emulsified in Freund’s incomplete adjuvant, a stimulator of the cellular immunity, was investigated. No protection against vertical transmission and cerebral infection in the pregnant mice and a very limited protective effect in the non-pregnant mice were observed. The vaccine induced a Th1-type immune response characterized by high IgG2a titres and strong IFN-γ expression, which appeared detrimental to pregnancy.

## 1. Introduction

*Neospora caninum* is an intracellular apicomplexan parasite, which is reported as a major cause of bovine abortion worldwide, and thus, neosporosis represents an important veterinary disease of high economic significance [1,2,3]. Vaccination against bovine neosporosis is a rational approach that appears to be the most cost-effective method for the control of the disease [4,5]. In order to confer optimal protection against neosporosis, a vaccine should be able to prevent both, the horizontal transmission from contaminated food or water and vertical transmission from an infected cow to its fetus [6,7]. Different strategies for the development of such a vaccine have been investigated [8,9,10,11]. The only commercialized vaccine, based on tachyzoite extract [12] did not achieve the required protection against vertical transmission [13] and was recently withdrawn from the market. However, more recent studies employing live vaccines showed promising results in cattle [14] and mice (reviewed in [10,15]). Our investigations focus on the use of recombinant proteins for vaccination against experimental challenge in the mouse model. Indeed, the mouse model provides valuable proof-of-concept data [16,17,18,19]. Studies employing different recombinant protein vaccine candidates have shown promising results in terms of limiting the parasite load and reducing the effects of the disease (reviewed in [15]). For instance, vaccination of mice with recNcMIC1 [20], recNcMIC3 [21] and recNcROP2 [22] conferred protection against cerebral infection and against vertical transmission when combined in a single dose vaccine [23]. Vaccination with a recombinant chimeric antigen composed of putative immunogenic domains of the three proteins (recNcMIC3-1-R) predicted *in silico* and emulsified in saponin adjuvants conferred a high level of protection against cerebral infection in non-pregnant mice [24], but did not prevent vertical transmission, nor cerebral infection in pregnant mice [25]. Comparison of antibody responses and cytokine data in pregnant and non-pregnant mouse models showed that a mixed Th1-/Th2-type immune response was associated with the protection observed in the non-pregnant mouse model, but a Th2-biased immune response was observed in the pregnant mice [24]. The use of saponin, a stimulator of humoral immunity, in the pregnant mouse model was thus thought to be detrimental to the Th1/Th2 balance that is obviously required for protection [25]. Thus, the use of an adjuvant that stimulates the cellular immunity, in order to counterbalance the inherent Th2-type biased immune response observed in the pregnant mice, may lead to protection. It was shown earlier that immunization of mice with antigens emulsified in complete or incomplete Freund’s adjuvant induced a Th1-type immune response by stimulating IFN-γ production [26,27]. In this paper, we report on the use of recNcMIC3-1-R emulsified in Freund’s incomplete adjuvant (FIA) for vaccination in the pregnant mouse model.

## 2. Results and Discussion

### 2.1. Cerebral Infection in Pregnant Mice

None of the formulations used produced visible lesions at the inoculation site or detectable adverse effects. All mice from the non-infected group survived without any clinical sign until the end of the experiment, and they tested all negative for *N. caninum* DNA in the brain (Table 1). All pregnant mice from the infected groups presented clinical signs of neosporosis and had to be killed before the end of the experiment, except for three out of eight mice in the group vaccinated with *N. caninum* crude extract (Table 1). 

**Table 1:** Neosporosis in experimentally infected mice.

Experimental groups	No. of mice		No. of sick mice (%)^a^		Mortality (%)^b^		*Nc* DNA in brain (%)^c^		Mean parasite load (±SD)^d^	
	P	NP	P	NP	P	NP	P	NP	P	NP
Non-infected control	9	11	0 (0)	0 (0)	0 (0)	0 (0)	0 (0)	0 (0)	0 (±0)	0 (±0)
FIA control	13	7	13 (100)	7 (100)	13 (100)	5 (71.4)	13 (100)	7 (100)	1446 (±184)	1054 (±204)
MIC3-1-R in FIA	8	12	8 (100)	9 (75)	8 (100)	7 (58.3)	8 (100)	12 (100)	1342 (±187)	851 (±364)
Crude extract in FIA	8	11	8 (100)	8 (72.7)	5 (62.5)	6 (54.5)	8 (100)	11 (100)	1179 (±400)	748 (±443)

^a^ Number of mice presenting at least one clinical sign of neosporosis (percentage); ^b^ Number of mice killed upon severe disease signs (percentage); ^c^ Number of dams tested positive for *N*; *caninum* DNA in the brain (percentage); ^d^ Mean parasite load per 80ng DNA in the positive mice (standard deviation); P = pregnant mice; NP = non-pregnant mice.

**Figure 1:**
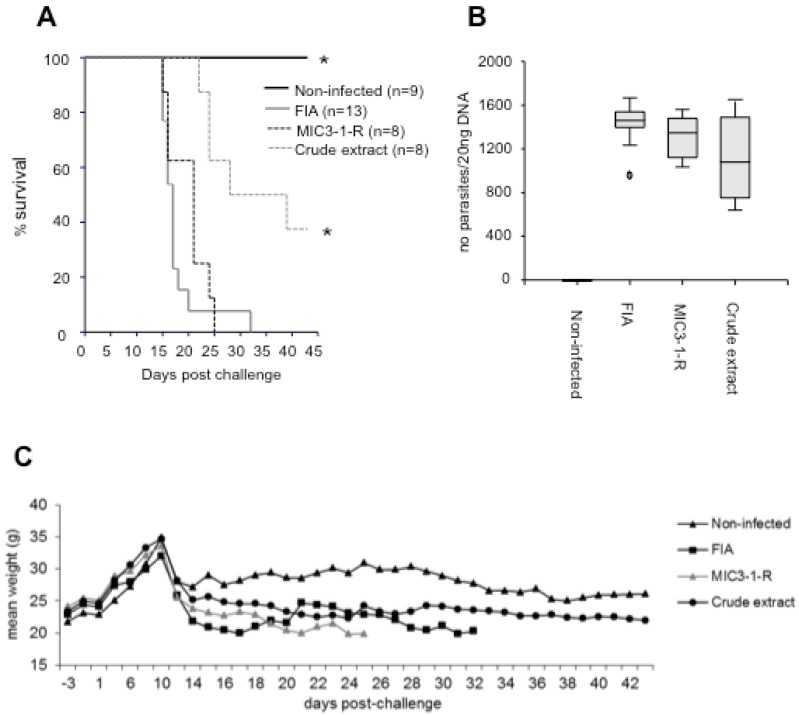
Effects of vaccination in pregnant mice. (**A**) Kaplan-Meier survival curves of mice; n indicates the number of mice per group; * indicates a significant difference compared to the FIA group *(P <* 0.01). (**B**) Cerebral parasite burden assessed by quantitative real-time PCR. The y-axis represents the number of parasites detected in 20ng of DNA extracted from brain tissue. No significant difference between the infected groups was observed. (**C**) Mean weight of the surviving mice per group.

The survival of the mice in the crude extract group was slightly higher than in the other infected groups, and the survival probability was significantly increased compared to each of the other infected groups (*P* < 0.01, Cox regression analysis) (Figure 1A). However, all mice from the infected groups tested positive for *N. caninum* DNA in the brain (Table 1, Figure 1B), and no significant difference between the parasite loads was observed between the groups. After delivery, the weight of the non-infected mice was significantly higher than the weight of all infected mice until the end of the experiment (*P* < 0.01, Mann-Whitney U-test) (Figure 1C). Significant differences between the crude extract group and the other infected groups were only observed at particular time points during the experiment (data not shown).

### 2.2. Cerebral Infection in Non-pregnant Mice

**Figure 2:**
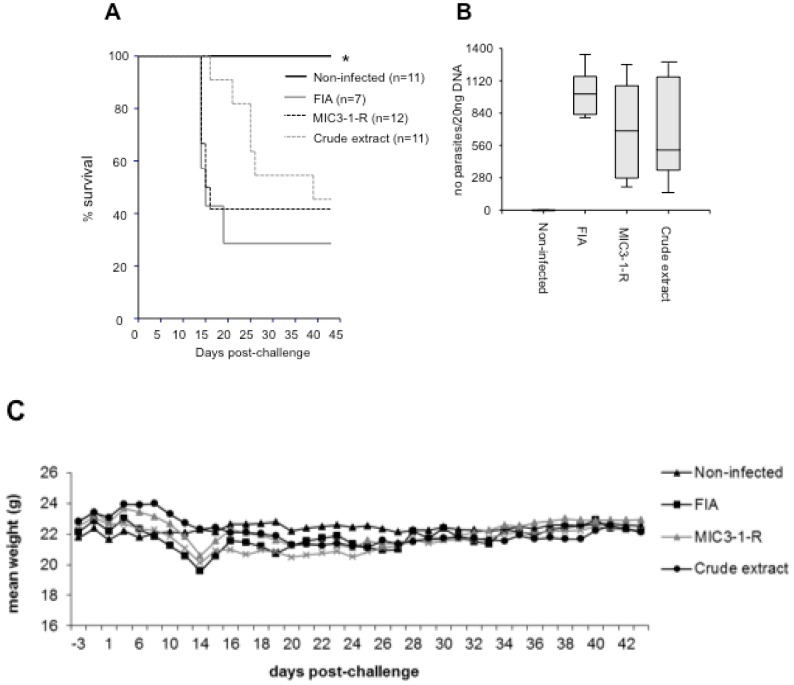
Effect of vaccination in non-pregnant mice. (**A**) Kaplan-Meier survival curves of mice; n indicates the number of mice per group; * indicates a significant difference compared to the FIA group *(P <* 0.01). (**B**) Cerebral parasite burden assessed by quantitative real-time PCR. The y-axis represents the number of parasites detected in 20 ng of DNA extracted from brain tissue. No significant difference between the infected groups was observed. (**C**) Mean weight of the surviving mice per group.

Non-pregnant mice presenting clinical signs of neosporosis were observed in all infected groups. However, a minority of mice from each vaccinated group did not present clinical signs and almost half of the mice from each of these groups survived until the end of the experiment (Table 1, Figure 2A). The mortality rate of the mice in the two vaccinated groups was lower than in the FIA group (Table 1), although the survival curves were not statistically different from the adjuvant control group (Log-rank test) (Figure 2A). All mice from the infected groups were nonetheless positive for *N. caninum* DNA in the brain (Table 1), but the parasite load in each infected group was significantly lower than in the respective group in the pregnant mice (*P* < 0.01 for the FIA and MIC3-1-R groups; *P* < 0.05 for the crude extract group, Mann-Whitney U-test) (Figure 2B). However, no significant difference between the vaccinated groups and the adjuvant control group was observed. No significant difference between the mean weights of the survivors at the end of the experiment was observed (Figure 2C). 

### 2.3. Vertical Transmission

No significant difference in the fertility rate was observed between the groups. However, the litter size in the non-infected group was significantly bigger than in all infected groups (*P* < 0.05, Mann-Whitney U-test), and the neonatal mortality was significantly lower in the non-infected group (*P* < 0.05, Mann-Whitney U-test) (Table 2). 

**Table 2:** Effects of *N. caninum* infection of pregnant mice on offspring survival in the different experimental groups.

Experimental groups	Fertility (%)^a^	No. of pups^b^	Average litter size (+/- SD)	Neonatal mortality (%)^c^	Postnatal mortality (%)^d^
Non-infected control	9/20 (45)	60	6.7 (1.7)	1 (1.7)	0/59 (0)
FIA control	13/20 (65)	64	4.9 (1.6)	13 (20.3)	51/51 (100)
MIC3-1-R in FIA	8/20 (40)	37	4.6 (1.4)	10 (27.0)	27/27 (100)
Crude extract in FIA	8/19 (42.1)	37	4.6 (1.7)	7 (18.9)	28/30 (93.3)

^a^ Number of pregnant mice / number of mice in the group (%); ^b^ Total number of pups born (alive or dead) from the whole group; ^c^ Number of pups born dead or that died within the 3 first days post partum (percentage); ^d^ Number of pups dead from day 4 to 31 p.p. / no. of pups alive at day 4 p.p. (percentage).

**Figure 3:**
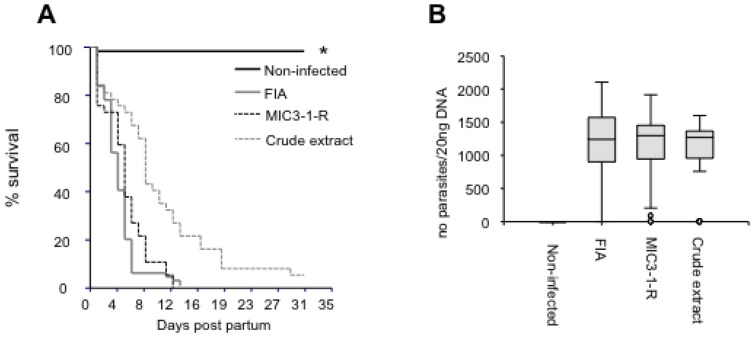
Effects of vaccination in offspring. (**A**) Kaplan-Meier survival curves of the pups; n indicates the overall number of pups per group; * indicates a statistically significant difference compared to the FIA group *(P <* 0.01). (**B**) Cerebral parasite burden assessed by quantitative real-time PCR in the pups. No significant difference between the infected groups was observed. The y-axis represents the number of parasites detected in 20 ng of DNA extracted from brain tissue.

All pups from the non-infected group that were alive on day 4 post-partum survived until the end of the experiment (Figure 3A), and tested negative for *N. caninum* DNA in the brain (Table 3). The survival of the pups from all infected groups was similar except for a minority of pups that survived longer in the crude extract group, although the survival curves were not significantly different (Log rank test) (Figure 3A). The number of dead neonatal mice testing positive for *N. caninum* DNA in the brain in the crude extract group was significantly lower than in the FIA group (*P* < 0.01, Chi-square test) (Table 3). All pups from each infected group died or had to be killed before the end of the experiment upon severe symptoms or euthanasia of the dam, except for two pups in the crude extract group. 

All pups tested positive for *N. caninum* DNA in the brain, and no significant difference in the parasite load of the infected pups was observed (Kaplan-Meier multiple comparison test) (Figure 3B).

**Table 3:** Detection of *N. caninum* infection in offspring mice of the different experimental groups.

Experimental groups	*Nc* DNA positive pups				Mean parasite load (±SD)^b^
	Neonatal deaths	Postnatal deaths	Survivors	Total	
Non-infected control	0/1 (0)	0/0 (0)	0/59 (0)	0/60 (0)	0 (±0)
FIA control	8/9 (88.9)	48/48 (100)	0/0 (0)	56/57 (98.2)	1277 (±457)
MIC3-1-R in FIA	4/7 (57.1)	25/25 (100)	0/0 (0)	29/32 (90.6)	1262 (±409)
Crude extract in FIA	1/7 (14.3)	25/25 (100)	2/2 (100)	28/34 (82.4)	1276 (±192)

### 2.4. Humoral Immune Response

**Figure 4:**
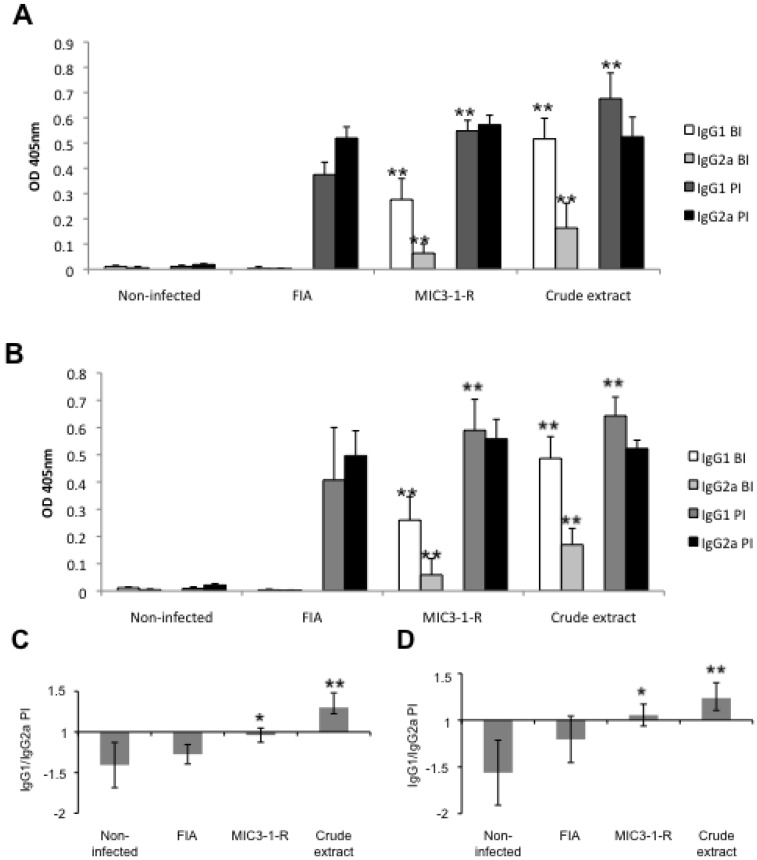
Humoral immune responses measured by ELISA employing mice sera taken after vaccination, 7 days after mating prior to challenge infection (BI) and after challenge, at time of euthanasia (PI). (**A**) and (**B**) shows IgG1 and IgG2a antibody responses against *N. caninum* crude extract for pregnant and non-pregnant mice respectively. Values are given as OD405nm ± SD. (**C**) and (**D**): ratios of IgG1/IgG2a post-infection for pregnant and non-pregnant mice respectively. Differences in antibody response were statistically significant: * *P* < 0.01, ** *P* < 0.001

Antibody-reactivity against crude *N. caninum* extract was assessed by ELISA. Similar patterns were observed in the pregnant (Figure 4A) and in the non-pregnant mice (Figure 4B). In the pre-immune sera, as well as in the pre-infection and post-infection sera of the non-infected group, specific IgG1 and IgG2a levels were close to zero. In the FIA group, both IgG1 and IgG2a levels remained low after vaccination and were only elevated after challenge infection. After vaccination with recNcMIC3-1-R and crude *N. caninum* lysate, elevated levels of specific IgG1 and IgG2a were observed. These levels were all significantly higher than the respective levels in the FIA group (*P* < 0.001, Mann-Whitney U-test). After infection, both IgG1 and IgG2a levels were increased in each vaccinated groups, and IgG1 levels in the MIC3-1-R and crude extract group were significantly higher than in the FIA group (*P* < 0.001, Mann-Whitney U-test), while IgG2a levels reached a similar level in each group. The ratio IgG1/IgG2a in the post-infection sera was significantly higher in the MIC3-1-R group and in the Crude extract group compared to the FIA group (*P* < 0.01 and *P* < 0.001 respectively, Mann-Whitney U-test). The ratios were similar in the pregnant (Figure 4C) and in the non-pregnant mice (Figure 4D). The ratio IgG1/IgG2a in the MIC3-1-R group was below 1 in the pregnant mice and over 1 in the non-pregnant mice, but the difference was not significant.

### 2.5. Cytokine Responses

IFN-γ, IL-4, IL-10 and IL-12(p70) levels in pre-immune, pre-infection and post-infection sera were measured in pools from each experimental group. Low levels of cytokines were detected in all pre-immune sera (data not shown), as well as in all sera from the non-infected group. 

**Figure 5:**
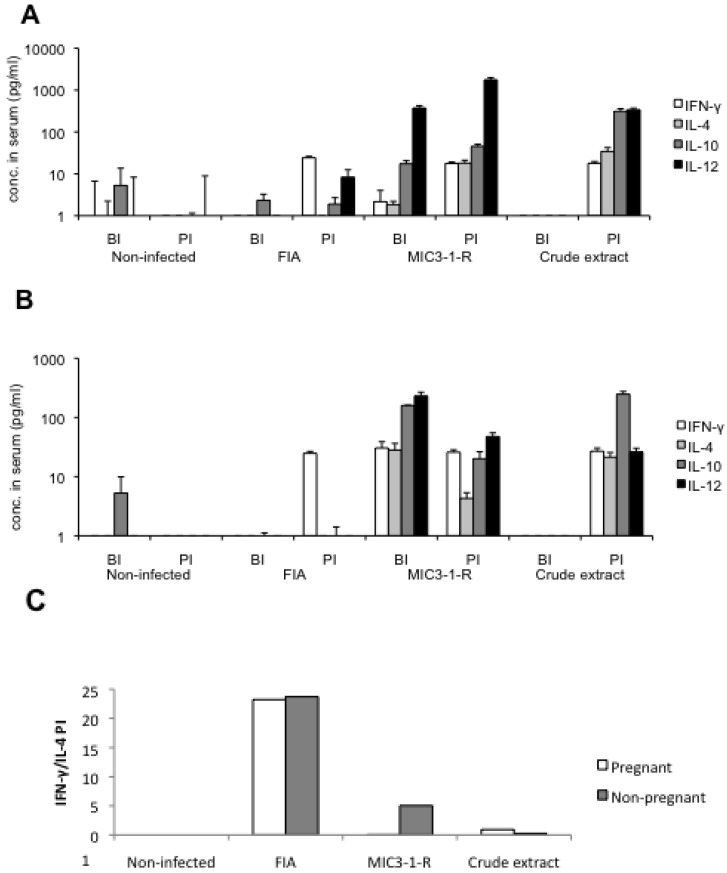
Cytokines analysis in sera taken after vaccination, prior to challenge infection (BI) and after challenge at the time of euthanasia (PI). The sera of all mice per group were pooled and analysed in triplicates. (**A**) and (**B**) represents the concentrations of IFN-γ, IL-4, IL-10 and IL-12 in pg/mL measured in each group for the pregnant mice and non-pregnant mice respectively. Values below the detection limit were set to 1 and the arbitrary value recommended by the kit manufacturer – 1 was subtracted from measurements. Error bars represent the standard deviation of the triplicates. (**C**) Ratios of IFN-γ/IL-4 after challenge infection.

In the FIA group, all cytokine levels were low in the pre-infection sera. IFN-γ level was increased up to 10 fold in the post infection sera of the pregnant and non-pregnant mice and IL-12 was slightly increased in the non-pregnant mice (Figure 5A,B). 

In the MIC3-1-R group, all cytokine levels in the pre-infection sera were elevated compared to non-infected and FIA immunized mice. While they were increased after challenge infection in the pregnant mice, they were all reduced in the non-pregnant ones. Post-infection IL-4, as well as IL-10 and IL-12 levels were much higher than in the FIA group, while IFN-γ was only slightly higher (Figure 5A,B).

Mice vaccinated with the crude tachyzoite lysate showed low levels of cytokines after vaccination and increased levels of all cytokines after challenge infection. The relative elevation of the cytokines post-infection was similar to that seen with the MIC3-1-R group. 

However, the post-infection IFN-γ/IL-4 ratio of all vaccinated group was equal or higher than 1 in both the pregnant and non-pregnant mice (Figure 5C). Moreover, this ratio was higher in the FIA group than in the MIC3-1-R and crude extract groups.

### 2.6. Discussion of Results

An efficient vaccine against bovine neosporosis has to prevent both horizontal and vertical transmission. Thus, studies in the animal model should focus on the outcomes of protection in both pregnant and non-pregnant animals. Among the vast number of investigations conducted in the mouse model, only few reported on protection against vertical transmission (reviewed in [15]). Indeed, as we previously showed, the same recombinant chimeric vaccine (recNcMIC3-1-R), which was protective in the non-pregnant mouse model [24] did not prevent the vertical transmission or the cerebral infection in pregnant dams [25]. The different outcomes of protection were associated with different cytokine profiles seen in pregnant and non-pregnant mice. A mixed Th1/Th2-type immune response was associated with protection in the non-pregnant mice while the lack of protection in the pregnant mice was associated with a Th2-type biased immune response [25]. As previously demonstrated, the choice of adjuvant is crucial to obtain the type of immune response sought [27,28]. In this study, we aimed to counterbalance the humoral immune response occurring during pregnancy by stimulating the cellular immune response with a Th-1 stimulator, namely FIA. Besides recNcMIC3-1-R, we included a crude antigen extract group due to its known partial efficacy against vertical transmission in mice [25,29] and cattle [13,30]. 

The vaccine composed of recNcMIC3-1-R induced a Th1-type biased immune response characterised by high IgG2a levels and an IgG1/IgG2a ratio close to 1 in both the pregnant and non-pregnant mice. High IFN-γ levels were observed in the post-infection sera. However, the IFN-γ/IL-4 ratio was higher in the non-pregnant mice than in the pregnant mice. Unfortunately, this vaccine formulation did not result in a balanced Th1/Th2 response, but in a strong Th-1-type immune response. This did not increase the protection against vertical transmission or cerebral infection in the pregnant mice and also decreased the protection against cerebral infection in the non-pregnant mice.

Interestingly, even if the protection against vertical transmission was lost when employing FIA as adjuvant, the protection achieved in the mice vaccinated with the crude extract group was still better than in the MIC3-1-R group. This response was associated with a mixed Th1-Th2-type immune response. The reduction of protection in this group compared to the formulation in saponin adjuvant may be explained by the lower IFN-γ expression, which appeared to be crucial for the control of the parasite replication *in vitro* [30] and in the mouse model [31].

The need of the appropriate Th1/Th2 balance for protection was already demonstrated in previous studies [32,33,34]. This is important considering that a Th2-type biased response was mainly associated with the vertical transmission [35,36] and exacerbation of the disease [37,38]. Indeed, it was recently demonstrated that *N. caninum* tachyzoites may infect dendritic cells and disseminate intracellularly across the placenta and the blood-brain barrier, thus, avoiding direct contact with serum antibodies [39]. On the other hand, a strong Th1-type immune response appeared to cause fetal death in cattle [40], and be detrimental in the mouse model [41,42,43,44]. Probably other important, but still under recognized, factors are also necessary for protection against neosporosis. Thus, new vaccination strategies should focus on wider immunological aspects, alternative ways of vaccine formulations and delivery. Indeed, Debache *et al.* [45] showed different outcomes of protection between intraperitoneal and intra-nasal vaccinations. Recently, Debache and Hemphill [46] also demonstrated high level protection against experimental challenge in mice by intra-cisternal vaccination. Despite the fact that the vaccine elicited high IgG2a and IFN-γ expression, which could be detrimental to the fetus, the outcome of the immune response may be different due to the immunomodulation that occurs during pregnancy.

## 3. Experimental Section

Unless otherwise stated, all cell culture reagents were supplied by Invitrogen (Paisly, UK) and chemicals were purchased from Sigma (St. Louis, MO, USA).

### 3.1. Culture and Purification of Parasites

*Neospora caninum* tachyzoites of the Nc-1 isolate [47] were maintained by serial passages in Vero cells using standard procedures [24]. Tachyzoites were harvested by repeatedly passaging infected cells through a 25 G-needle, and liberated parasites were purified on Sephadex G25 PD-10 columns (GE Healthcare, Buckinghamshire, England) [48]. Parasites were counted in a Neubauer chamber using trypan blue stain exclusion. For challenge infections, parasite numbers were adjusted to 2 × 10^7^/mL in RPMI 1640 medium and tachyzoites were used immediately for infection experiments (100 µL/mouse).

### 3.2. Preparation of *N. caninum* Crude Protein Extract

*N. caninum* tachyzoites were purified as described in the previous section and adjusted to 1 × 10^8^ parasites/mL in sterile PBS. They were then disrupted by performing 4 freeze/thaw cycles using a dry-ice/methanol bath and a 37 °C water bath followed by sonication for 5 × 1 min at 57 W (Branson Sonifier cell disruptor, Skan AG, Allschwill, Switzerland). The parasites were observed microscopically to ensure that complete disruption had occurred, and the protein concentration was measured using the Protein Assay Dye Reagent Concentrate (Bio-Rad, Hercules, CA, USA) and acetylated BSA as a standard. Just prior to vaccination, the lysate was vortexed and sonicated until a homogenous suspension was obtained.

### 3.3. Preparation of recNcMIC3-1-R

The recombinant chimeric protein recNcMIC3-1-R was obtained as described in [24]. Briefly, *in silico* predicted putative immunogenic epitopes of NcMIC1, NcMIC3 and NcROP2 were amplified from *N. caninum* cDNA and assembled as a single gene. RecNcMIC3-1-R was expressed in One Shot^®^ BL21 (DE3) Chemically Competent *E. coli* (Invitrogen) and purified by Ni^2+^-chelate chromatography under denaturing conditions. After dialysis against 1 g/L ammonium bicarbonate the protein was dried in Speedvac and resuspended in PBS. The protein concentration was measured using the Protein Assay Dye Reagent Concentrate (Bio-Rad, Hercules, CA, USA) and acetylated BSA as a standard.

### 3.4. Vaccination Trial, Clinical Monitoring and Sample Collection

Six weeks old BALB/c mice were purchased from Charles River Laboratories (Sulzfeld, Germany) and maintained under conventional day/night cycle housing conditions as required by the animal welfare legislation of the Swiss Veterinary Office. They were used for experimentation after two weeks of acclimatization. Females were randomly allocated into four groups of twenty animals each and one group of 19 mice. Each group was vaccinated three times by intraperitoneal (i.p.) injection at 2 weeks interval as follows: group 1 (non-infected) was injected with 100μl PBS; group 2 (FIA) was injected with 100 μL Freund’s incomplete adjuvant (adjuvant control); group 3 (MIC3-1-R) was injected with 10 μg recNcMIC3-1-R emulsified in FIA; group 4 (crude extract) was vaccinated with 10μg *N. caninum* lysate (equivalent to approximately 1 × 10^6^ parasites). Just prior to use, freshly purified recNcMIC3-1-R, as well as the crude protein extract were emulsified at the appropriate concentrations in Freund’s incomplete adjuvant (100 μL/injection). Two weeks after the third immunization, synchronization of oestrus by the Whitten effect [49], followed by mating for three nights was performed. Seven days after mating (day 7–9 of pregnancy), all mice from groups 2–4 were challenged i.p. with 2 × 10^6^ freshly purified Nc-1 tachyzoites in 100 μL RPMI 1640 medium. The mice from group 1 received 100 μL RPMI 1640 medium. The pregnant mice were then housed separately to rear their pups, and all animals were checked for clinical signs on a daily basis until day 43 post challenge. Data on the fertility rate (percentage of pregnant mice/group), litter size, neonatal mortality (pups born dead or that died within the three first days post-partum) and postnatal mortality (pups dead between day 4 and 31 post-partum) were collected. Dams’ weights were monitored by daily measurement from day 2 before challenge until the end of the experiment, and pups were weighed from day 14 post-partum onward. Mice exhibiting clinical signs of neosporosis (ruffled coat, apathy, neurological and walking disorders), were euthanized at the onset of these signs. On day 43 post challenge, all surviving dams and pups were sacrificed by CO_2_ euthanasia. Upon euthanasia, blood was drawn by cardiac puncture and sera were frozen at −80 °C. Brains were collected using individual sterile instruments and were immediately frozen at −20 °C. 

### 3.5. Cerebral Parasite Burden

*Neospora*-specific quantitative real-time PCR to determine the number of parasites present in the cerebral tissue was performed as described by Müller *et al.* [50]. DNA extraction from brain tissue was performed as described [22], and DNA concentrations were adjusted to 20ng/μL with sterile DNase-free water. Quantitative real-time PCR was performed using the Light Cycler™ Instrument (Roche Diagnostic, Basel, Switzerland). The parasite counts were calculated by interpolation from a standard curve with DNA equivalents from 1000, 100 and 10 parasites included in each run [25].

### 3.6. Serology

Individual sera taken at three distinct time points in the dams (pre-immune, post-immunization prior to infection (day 9 after the last immunization boost), and post-infection (at the time of euthanasia) were analyzed for *N. caninum* specific IgG1 and IgG2a by ELISA coated with *N. caninum* lysate prepared as described in section 2.2. The ELISAs were performed with a 1:100 dilution of the sera [25]. Serum from a mouse experimentally infected with Nc-1 that had reacted positively against *N. caninum* lysate in ELISA and serum from a non-infected mouse were used as positive and negative control, respectively.

### 3.7. Serum Cytokine Levels

Serum levels of IFN-γ, IL-4, IL-10 and IL-12(p70) were measured by means of multiplex bead immunoassay using the MILLIPLEX^®^MAP Mouse Cytokine/Chemokine kit (Millipore Corp. Billerica, MA, USA). Undiluted post-immunization and post-infection sera were pooled per group and analyzed in triplicate according to the manufacturer’s protocol. Microtitre filter plates were run on Luminex instruments (Bio-Plex™ 200 system, Bio-Rad), and cytokine concentrations were calculated with the Bio-Plex Manager software [25]. When cytokine concentration was below the detection limit, an arbitrary value corresponding to the detection limit of undiluted samples (provided by the kit manufacturer) was used for analysis. Arbitrary values corresponded to: 1 pg/mL for IFN-γ; 1 pg/mL for IL-4; 1.5 pg/mL for IL-10; 6 pg/mL for IL-12.

### 3.8. Statistical Analysis

Dam survival and pup mortality were analyzed according to Kaplan-Meier, and the survival curves between groups were compared with the logrank test followed by regression coefficient analysis. Litter size, neonatal mortality, weight, cerebral parasite burden, as well as serological data were compared using Kruskal-Wallis one-way ANOVA followed by the Kruskal-Wallis multiple comparison Z-value test. The *P*-value between two significantly different groups was calculated by Mann-Whitney U test. The fertility rates, proportion of animals with clinical signs, and data on *N. caninum* DNA positive animals were organized in a contingency table and compared by a Chi-square test.

## 4. Conclusions

In summary, the use of FIA combined with the recombinant antigen recNcMIC3-1-R for the vaccination of experimentally infected mice was not protective against vertical transmission or against cerebral infection in pregnant and non-pregnant mice. Although the immune responses observed were different from those obtained with the vaccines formulated in saponin adjuvant, the shift toward a Th-1-type immune response may have been too strong, and the sensitive Th1/Th2 balance needed was not reached. This study demonstrates once again the difficulty to achieve protection against experimental neosporosis during pregnancy, either through a Th1- or a Th2-type immune response. Further recombinant vaccine development should focus on the finding of the appropriate Th1/Th2 balance and other aspects of the immune response that were not or poorly investigated so far. The use of expression systems that allow to fuse antigens of interest to a suitable Toll-like receptor ligand, as described e.g., by Basto *et al.* [51] could provide the means of modulating the immune response in order to limit the consequences of *N. caninum* infection.
